# Allergic rhinitis: evidence for impact on asthma

**DOI:** 10.1186/1471-2466-6-S1-S4

**Published:** 2006-11-30

**Authors:** Mike Thomas

**Affiliations:** 1 Department of General Practice and Primary Care, University of Aberdeen, Foresterhill Health Centre, Westburn Road, Aberdeen AB25 2AY, UK

## Abstract

**Background:**

This paper reviews the current evidence indicating that comorbid allergic rhinitis may have clinically relevant effects on asthma.

**Discussion:**

Allergic rhinitis is very common in patients with asthma, with a reported prevalence of up to 100% in those with allergic asthma. While the temporal relation of allergic rhinitis and asthma diagnoses can be variable, the diagnosis of allergic rhinitis often precedes that of asthma. Rhinitis is an independent risk factor for the subsequent development of asthma in both atopic and nonatopic individuals. Controlled studies have provided conflicting results regarding the benefits for asthma symptoms of treating comorbid allergic rhinitis with intranasal corticosteroids. Effects of other treatments for comorbid allergic rhinitis, including antihistamines, allergen immunotherapy, systemic anti-IgE therapy, and antileukotriene agents, have been examined in a limited number of studies; anti-IgE therapy and antileukotriene agents such as the leukotriene receptor antagonists have benefits for treating both allergic rhinitis and asthma. Results of observational studies indicate that treating comorbid allergic rhinitis results in a lowered risk of asthma-related hospitalizations and emergency visits. Results of several retrospective database studies in the United States and in Europe indicate that, for patients with asthma, the presence of comorbid allergic rhinitis is associated with higher total annual medical costs, greater prescribing frequency of asthma-related medications, as well as increased likelihood of asthma-related hospital admissions and emergency visits. There is therefore evidence suggesting that comorbid allergic rhinitis is a marker for more difficult to control asthma and worsened asthma outcomes.

**Conclusion:**

These findings highlight the potential for improving asthma outcomes by following a combined therapeutic approach to comorbid allergic rhinitis and asthma rather than targeting each condition separately.

## Background

Many patients with asthma, particularly those with allergic asthma, also have allergic rhinitis (AR). The mucosa of the upper and lower airways is continuous, and the type of inflammation in AR and asthma is very similar, involving T helper type 2 cells, mast cells, and eosinophils, as reviewed in the following paper in the present supplement [1]. Allergic rhinitis is now subdivided into intermittent AR (symptoms <4 days per week or for <4 weeks) and persistent AR (symptoms >4 days per week or for >4 weeks), rather than the previous subdivisions of seasonal and perennial AR, and is further characterized according to severity as mild or moderate/severe [2]. Treatment of AR is based on the subdivision and the severity.

Current evidence indicates that comorbid AR may have clinically relevant effects on asthma. The database of evidence is still relatively small, and much of the data come from observational studies. Moreover, the definitions of AR and asthma differ among studies. Nonetheless, the association between AR and asthma was sufficiently apparent that, in 1999, a workshop was held at the World Health Organization to develop evidence-based guidelines for managing rhinitis and to highlight the impact of AR on asthma. This global program – entitled the Allergic Rhinitis and its Impact on Asthma initiative – is ongoing, in collaboration with the World Health Organization, to translate evolving science into clinical recommendations for managing and preventing rhinitis, to better assess the interactions between rhinitis and asthma, to increase awareness of rhinitis, and to make effective treatment of rhinitis available worldwide [2].

This paper reviews the current evidence indicating that comorbid AR may have clinically relevant effects on asthma. Nonallergic asthma and rhinitis appear also to be associated, although these disorders are less well understood than their allergic counterparts [2]. The designation of 'nonallergic' is applied when the allergy examination, including history, skin-prick testing, and serum-specific IgE measurements, is negative.

## Asthma and allergic rhinitis often occur concomitantly

AR is very common in patients with asthma [2], with a reported prevalence of up to 100% in those with allergic asthma [3]. In a recent review examining prevalence studies of comorbid AR published from 1983 to 2004 [4], the point prevalence of AR ranged from 24% to 94% and the lifetime prevalence ranged from 50% to 100% among adults with asthma in Europe and in the United States. These findings have been corroborated in more recent studies from Europe and from Japan [5,6].

The variability in the reported prevalence of comorbid AR in the studies reviewed was attributable in part to differences in diagnostic criteria and study design [4]. Geographical differences may exist also: in the only study from Asia meeting the criteria for the present review [7], the prevalence of comorbid AR in people with asthma in rural China was lower (6%). Among school-age children surveyed in the International Study of Asthma and Allergy in Children, there are striking variations in the prevalence of asthma and allergic rhinoconjunctivitis symptoms recorded among different centers worldwide; nonetheless, significant correlations (*r *= 0.75, *P *< 0.0001) are noted between the prevalence of asthma and allergic rhinoconjunctivitis symptoms [8,9].

Several medical database studies have examined the association between AR and asthma [10-13]. In the Rochester, Minnesota, USA study [10], AR was most common (59% prevalence) among people whose asthma was diagnosed before age 25, and AR was relatively uncommon (15%) among those whose asthma was diagnosed after age 40 (9% of all subjects). The overall prevalence of documented AR in this study was 52% among the 1,245 patients with asthma. In the UK Medi-Plus general practice database studies [11,12], concomitant AR was documented in medical records of only 17% of 27,303 patients aged 16–55 years with asthma and in 20% of 9,522 children with asthma. Similarly, of 2,961 children in the national database in Norway with at least one hospital admission for asthma over a 2-year period, 27% had a documented history of AR [13]. It is possible that the prevalence of comorbid AR among patients in these retrospective studies was underestimated because the diagnosis of AR was restricted to that recorded in medical records. Many people with AR self-manage the condition with over-the-counter products, do not seek a physician's help, or indeed do not recognize AR as a condition needing treatment [5].

Conversely, asthma is often present in patients with AR. Linneburg and coworkers [3] reported asthma in 25% of patients with AR who were pollen-sensitive and in 50% of those AR patients who were mite-sensitive or animal-sensitive. Greisner and coworkers [14] report a history of asthma among 21% of former college students with a cumulative history of AR over 23 years of follow-up.

In the European Community Respiratory Health Survey, an association between asthma and rhinitis was observed even in nonatopic individuals [15]. This finding implies that the relationship cannot be fully explained by shared risk factors and supports the hypothesis that upper-airway disorders may directly affect the lower airways.

## Impact of allergic rhinitis on asthma

While the temporal relation of AR and asthma diagnoses can be variable, the diagnosis of AR often precedes that of asthma [14,16]. In fact, rhinitis is an independent risk factor, both in atopic and nonatopic individuals, for the subsequent development of asthma [16-20].

Bronchial hyperresponsiveness is common in people with AR, even if they have no asthma symptoms, and asymptomatic airway hyperresponsiveness is associated with increased risk for developing asthma [21,22]. In one study, 40% of patients with AR showed hyperresponsiveness to methacholine challenge; those showing hyperresponsiveness were more likely to develop asthma over the following 4–5 years [23]. Moreover, among patients with seasonal AR, response to a nonspecific bronchial provocation test increases during the pollen season [24]. Among patients who had shown exacerbation of asthma symptoms in conjunction with the onset of seasonal AR, nasal allergen challenge resulted in increased nonspecific bronchial responsiveness [25]. As further evidence of a pathophysiological link between upper and lower airways, segmental bronchial provocation in patients with AR but no asthma results in allergic inflammatory changes in the nose [26].

Comparisons of the clinical characteristics of asthma in patients with and without AR are few [27,28]. One small study found that patients with perennial AR are more at risk of developing bronchial symptoms than healthy control subjects [29]. Asthma exacerbations frequently occur coincident with the worsening of nasal symptoms [6]. In another study, asthma severity among atopic asthmatic patients was less in those with nasal symptoms than in those without nasal symptoms, whereas asthma severity among nonatopic asthmatic patients was greater in those with nasal symptoms than in those without nasal symptoms [28]. Conversely, among patients with severe, corticosteroid-dependent asthma, sinonasal involvement is almost universal [30].

Several retrospective cost-of-illness studies have explored healthcare costs for patients with asthma who have comorbid AR [10-13,31]. In two US studies, total annual medical costs were appreciably higher for patients with comorbid AR relative to those with only asthma and no AR [10,31]. In addition, in one study the presence of AR was associated with higher costs and greater prescribing frequency of asthma-related medications [31]. In Rochester, Minnesota, total costs were 46% higher overall with comorbid AR; however, when age groups were examined separately, significantly higher costs with comorbid AR were not evident beyond age 25 [10]. Moreover, for patients from 55 to 64 years old, annual medical costs were significantly higher for those with only asthma.

In the UK Medi-Plus general practice database studies, the presence of physician-recorded comorbid AR was associated with a 50% increase in rate of hospitalization for asthma among adults and a 250% increase in rate of hospitalization for asthma among children during a 12-month follow-up period [11,12]. The number of asthma-related visits to a general practitioner and the asthma drug costs were also significantly higher among both adults and children with documented comorbid rhinitis. Similarly, for Norwegian children with asthma, the presence of comorbid rhinitis was associated with increased likelihood of asthma-related hospital readmissions and greater total hospital days [13]. In a recent German study of patients with moderate-to-severe asthma, the annual cost of illness increased for both children and adults with the severity of asthma and the presence of concomitant seasonal AR [32].

**Figure 1:**
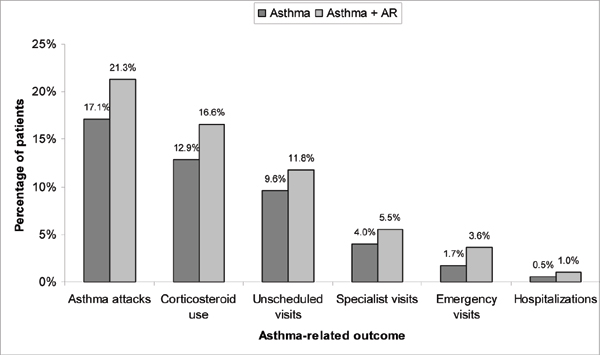
Asthma-related resource use and asthma attacks according to the presence of concomitant allergic rhinitis. In a post-hoc analysis of the Investigation of Montelukast as a Partner Agent for Complementary Therapy trial, rates of asthma attacks and emergency room visits were significantly higher among patients with comorbid asthma and allergic rhinitis (AR). Adapted with permission from Bousquet and coworkers [34].

In the controlled trial setting, a post-hoc analysis of the Investigation of Montelukast as a Partner Agent for Complementary Therapy trial indicates that, in addition to greater use of healthcare resources, outcomes are worse for patients with comorbid rhinitis and asthma [33,34]. This trial compared the addition of montelukast therapy or salmeterol therapy over 12 months for 1,490 adults with mild-to-moderate asthma not controlled by inhaled fluticasone alone [33]. Self-reported comorbid AR was present in 60% of patients enrolled in this study; these patients tended to be younger and to have less severe asthma than those with asthma alone. Nonetheless, significantly more patients with comorbid AR experienced emergency room visits and asthma attacks; the frequencies of hospitalizations and unscheduled or specialist visits were greater among those with concomitant AR but did not differ significantly between the two groups (Figure 1) [34].

## Treatment of allergic rhinitis: effect on asthma-related outcomes

The evidence thus suggests that comorbid AR is a marker for more difficult to control asthma and for worsened asthma outcomes. This leads to the question of whether treating comorbid AR would produce better asthma-related outcomes in addition to the obvious benefits with regard to rhinitic symptoms. There is presently a paucity of data on this topic, and there is some inconsistency in reported outcomes with different AR treatment strategies.

Two small studies in the 1980s showed benefits of intranasal corticosteroids for asthma symptoms. In one study, considerable reductions in seasonal asthma symptoms were recorded among patients with concomitant AR who were treated with intranasal beclomethasone or flunisolide [35]. In the second study, cough and exercise-induced asthma symptoms were reduced among children with perennial AR treated with intranasal budesonide [36].

More recent studies have produced conflicting results regarding the effects of intranasal corticosteroids on the lower airways of patients with AR. Some of these studies have shown decreased bronchial hyperresponsiveness after treatment with intranasal corticosteroids [37-40], while other studies failed to show this [41-44]. One study reported positive effects of intranasal corticosteroids on symptoms of asthma but not on bronchial responsiveness [45], while another study showed no improvement in asthma symptoms but the effects on bronchial responsiveness were not measured [46]. It is important to note that all but one of these studies [46] enrolled small numbers of patients. In addition, study designs and patient characteristics, including age and the concomitant presence or absence of asthma, differed among the studies. Moreover, compared with newer intranasal corticosteroids, some of the older intranasal corticosteroids have higher oral and systemic bioavailability; this may account for effects on lower airways in some studies.

Effects of other treatments for comorbid AR, including antihistamines, allergen immunotherapy, systemic anti-IgE therapy, and antileukotriene agents, have been examined in a limited number of studies. Systemic effects of these treatments may play a role in effects on bronchial hyperresponsiveness and asthma symptoms. While antihistamines are not considered effective for treating asthma *per se*, results of some studies suggest that an oral antihistamine given to patients with comorbid AR and asthma can improve persistent asthma symptoms [47] and nonspecific bronchial hyperresponsiveness [48], as well as asthma symptoms during the pollen season [49]. Similarly, there is recent evidence that immunotherapy may clinically benefit lower airway function in patients with AR, although the results are not consistent and the study designs vary. Specific immunotherapy is reported to reduce bronchial hyperresponsiveness in patients with AR in some studies [50,51] but not in others [52,53]. In recent reports, treatment with allergen immunotherapy reduced the development of asthma in children and adults with AR [54,55]. Systemic anti-IgE therapy also shows promise for treating patents with comorbid asthma and AR, particularly those with disease at the moderate to severe end of the spectrum. Anti-IgE therapy with omalizumab improves symptoms, improves quality of life and reduces asthma exacerbations in patients with concomitant asthma and persistent AR [56].

Antileukotriene agents such as the leukotriene receptor antagonists have benefits for treating both AR and asthma. Philip and coworkers [57] report that montelukast therapy improved asthma outcomes as well as providing significant relief from symptoms of seasonal AR in a multicenter study of 831 adult patients with seasonal allergen sensitivity, with active symptoms of seasonal AR, and with active asthma. Moreover, in a post-hoc analysis of a randomized controlled trial comparing the addition of montelukast with doubling the dose of inhaled corticosteroid for patients whose asthma was uncontrolled on the standard dose of inhaled corticosteroid [58], outcomes were superior for the 216 patients with comorbid AR given montelukast than for the 184 patients with comorbid AR given a doubled dose of inhaled corticosteroid; this finding implies an additional benefit to asthma control from a systemic agent able to treat AR as well as asthma [59]. By contrast, the results of adding montelukast versus doubling inhaled corticosteroid were not different for the 497 patients with asthma alone [59].

In another recent study, Ragab and coworkers [60] report improved asthma symptoms and asthma control correlating with improved upper airway symptoms after either surgical or medical treatment of chronic rhinosinusitis for patients with comorbid asthma. Significant improvements in overall asthma control after either type of treatment modality were recorded; however, improvements were better maintained after medical therapy of rhinosinusitis, which consisted of a 12-week course of oral erythromycin, alkaline nasal douches, and intranasal corticosteroids.

Recently published observational data also support the concept that asthma outcomes are better, for both children and adults, when comorbid AR is treated [61-63]. Crystal-Peters and coworkers [61] evaluated data for almost 5,000 US patients aged 12–60 years with comorbid AR and asthma. They found for the three-quarters of patients who were receiving treatment for AR that the risk of an asthma-related event (hospitalization or Emergency Department visits) was one-half that for patients not receiving treatment for AR. Similarly, in an Australian managed care population of 14,000 patients older than 5 years of age with asthma, treatment of nasal conditions with intranasal corticosteroids substantially reduced the risk of an Emergency Department visit for asthma [62], although the methods of this study have been criticized as allowing an immortal time bias to potentially act as a confounding factor [64].

Corren and coworkers [63] conducted a nested case–control study of a US managed care population of patients aged 6 years and older. For those with concomitant asthma and AR, treatment with either nasal corticosteroids or second-generation antihistamines was associated with a significant reduction in risk of hospitalization for asthma. Patients receiving nasal corticosteroids also had a significantly lowered risk of asthma-related Emergency Room treatment [63].

## Conclusions and remaining questions

In summary, asthma and AR frequently occur concomitantly. The presence of AR often precedes the development of asthma and is a known risk factor for asthma. There is evidence that having comorbid AR is a marker for the presence of more difficult to control asthma and therefore greater use of resources for asthma. There are also strong indications from observational data that treating comorbid AR may result in better asthma outcomes.

Several questions remain to be answered by future studies. Is AR being diagnosed properly in patients with asthma, as recommended in the Allergic Rhinitis and its Impact on Asthma guidelines? When AR is diagnosed, are patients being treated in the best possible way? Indeed, what is the best way of treating AR when comorbidity exists, with a particular focus on the asthma outcomes?

At present, treatment typically follows a two-compartment model whereby asthma and rhinitis are each treated separately and often locally, or topically; treatment is administered seasonally for people with seasonal rhinitis. Asthma outcomes might improve for patients with comorbid AR and asthma if treatment was instead long term and followed a combined therapeutic approach for the two conditions.

## Abbreviations

AR = allergic rhinitis.

## Competing interests

MT has no shares in any pharmaceutical company. Either through his role at the University of Aberdeen or personally MT has received grants, honoraria or educational support from the following companies as well as the UK NHS R&D programme and Asthma UK: Altana, AstraZeneca, GlaxoSmithKline, Ivax, Merck, Sharp and Dohme, Novartis, Schering Plough, Trinity Pharmaceuticals, Viatris.
